# Matrix Production, Pigment Synthesis, and Sporulation in a Marine Isolated Strain of *Bacillus pumilus*

**DOI:** 10.3390/md13106472

**Published:** 2015-10-21

**Authors:** Blanda Di Luccia, Antonio Riccio, Adele Vanacore, Loredana Baccigalupi, Antonio Molinaro, Ezio Ricca

**Affiliations:** 1 Department of Biology, Federico II University, MSA, via Cinthia 21, 80126 Naples, Italy; E-Mails: blanda.diluccia@unina.it (B.D.L.); antonio.riccio.91@gmail.com (A.R.); lorbacci@unina.it (L.B.); 2 Department of Chemistry, Federico II University, MSA, via Cinthia 21, 80126 Naples, Italy; E-Mails: eleda-86@libero.it (A.V.); molinaro@unina.it (A.M.)

**Keywords:** SF214, microbial differentiation, exopolysaccharide

## Abstract

The ability to produce an extracellular matrix and form multicellular communities is an adaptive behavior shared by many bacteria. In *Bacillus subtilis*, the model system for spore-forming bacteria, matrix production is one of the possible differentiation pathways that a cell can follow when vegetative growth is no longer feasible. While in *B. subtilis* the genetic system controlling matrix production has been studied in detail, it is still unclear whether other spore formers utilize similar mechanisms. We report that SF214, a pigmented strain of *Bacillus pumilus* isolated from the marine environment, can produce an extracellular matrix relying on orthologs of many of the genes known to be important for matrix synthesis in *B. subtilis*. We also report a characterization of the carbohydrates forming the extracellular matrix of strain SF214. The isolation and characterization of mutants altered in matrix synthesis, pigmentation, and spore formation suggest that in strain SF214 the three processes are strictly interconnected and regulated by a common molecular mechanism.

## 1. Introduction

Members of the *Bacillus* and *Clostridium* genera are Gram-positive bacteria able to survive harsh environmental conditions by producing a metabolically quiescent and extremely resistant spore. This peculiar cell is characterized by a dehydrated cytoplasm surrounded by a series of protective layers: a peptidoglycan-like cortex and a proteinaceous coat [1]. Some species of the two genera also have an exosporium, an additional layer surrounding the coat [1]. When released in the environment, the spore survives long-term absence of water and nutrients and withstands extreme conditions that would be lethal for other cell types [2]. Spore formers have been isolated from many diverse habitats, including soils, marine environments, and the gut of various insects and animals [2,3]. Although metabolically quiescent, the spore senses the environment and when nutrients are present, it germinates, forming a cell able to grow and eventually to re-sporulate [4].

**Figure 1:**
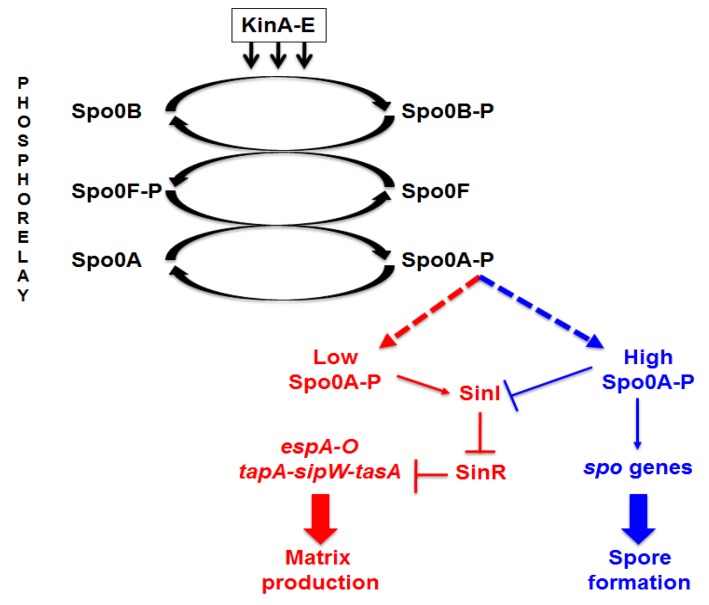
The matrix/sporulation switch in *B. subtilis.* Schematic diagram of the main factors involved in controlling the synthesis of matrix components and the entry into the sporulation cycle in *B. subtilis* [5,9,10].

Spore formation is induced by environmental conditions that do not allow cell growth, such as a blockage of DNA replication and a decline in available nutrients [5]. However, producing a spore is not the only possibility for cells unable to continue vegetative growth. In *Bacillus subtilis*, when planktonic cells reach the end of the exponential phase of growth they can follow alternative developmental pathways, with some cells producing a polymeric matrix rich in sugars and proteins and assembling into a multicellular biofilm and others entering the irreversible program of spore formation [6,7,8,9]. In a planktonic cell, population matrix and spore production are mutually exclusive cell fates [6,9] and both are bimodal processes in which cells follow either one or the other pathway [8,9]. Both processes are induced by a series of histidine kinases, KinA–E, which activate a phosphorelay that culminates in the phosphorylation and activation of the master transcriptional regulator Spo0A [10,11]. As summarized in Figure 1, when present in the cytoplasm at low concentration Spo0A–P upregulates SinI, an anti-repressor that antagonizes the biofilm repressor SinR [10]. Relief of SinR-mediated repression, then, causes the expression of genes coding for either structural components of the matrix or proteins needed for the synthesis/transport of matrix components [5,10]. High levels of Spo0A–P instead block matrix formation and activate the expression of early sporulation genes, thus inducing spore formation [5,10]. The key role of SinR for matrix production in *B. subtilis* is also suggested by its identification as a mutational target for fine-tuning of biofilm formation [12].

The rigid extracellular matrix produced by *B. subtilis* contains exopolymeric substances (EPS) and proteins [13]. The EPS structure has not been defined yet but it is known that its synthesis and secretion need the products of the *epsA–O* operon [5,9,10]. Mutants that do not express the *eps* operon produce an extremely fragile matrix, formed of only protein components [9,10]. Major protein components of the matrix are TapA and TasA, encoded, respectively, by the first and third gene of the three-gene operon *tapA-sipW-tasA* [9,10]. The second gene of the operon encodes SipW, a protein needed for TasA and TapA secretion. Outside the cell the amyloid-like TasA protein self-assembles into fibers that are anchored by TapA to the cell wall [9,10]. Matrix formation and the regulatory circuits linking it to sporulation have mostly been studied in *B. subtilis* [5,9,10] and only recently investigated in other *Bacillus* species [14,15,16].

We have previously reported that SF214, a pigmented strain of *Bacillus pumilus* isolated from a marine environment, [17], also produces a matrix [18]. Strain SF214 contains a TasA homolog [18], suggesting that the matrix of *B. pumilus* is similar to that of *B. subtilis*. In strain SF214 pigment synthesis, essential for cell resistance to hydrogen peroxide, only occurs in cells that do not form spores [18]. Some pigmented cells of strain SF214, however, also produce a matrix [18]. Here we analyzed the matrix of SF214 by performing a bioinformatic analysis of the genes involved in matrix formation, a chemical characterization of the carbohydrates of the matrix, and a physiological analysis of the growth conditions that favor matrix synthesis. In addition, we isolated and characterized nitrosoguanidine (NTG)-induced mutants of strain SF214 with altered pigment production and showed that they are also altered in matrix production and sporulation, confirming that a common regulatory circuit links the three processes.

## 2. Results and Discussion

### 2.1. Matrix Genes in B. pumilus ATCC 7061^T^ and SF214

**Figure 2:**
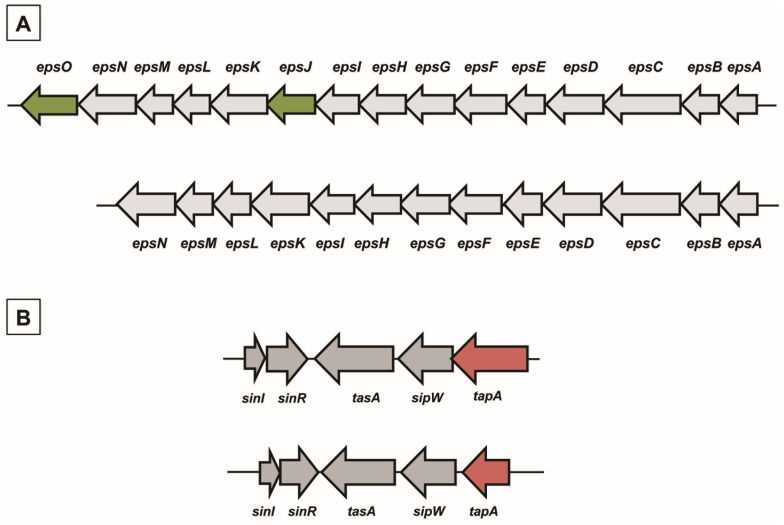
Chromosomal organization of genes involved in matrix synthesis in *B. subtilis* and *B. pumilus*. (**A**) The *epsA–O* operon in *B. subtilis* (top) and *B. pumilus* (bottom). In dark green are indicated two genes present in *B. subtilis* and lacking in *B. pumilus*. (**B**) Chromosomal organization of the *sinI–sinR* gene pair and the *tapA-sipW-tasA* operon in *B. subtilis* (top) and *B. pumilus* (bottom)*.* In red is the *tapA* gene, significantly smaller in *B. pumilus* than in *B. subtilis*.

In *B. subtilis* the main matrix genes are clustered in the *epsA-O* and *tapA-sipW-tasA* operons [10]. We analyzed the genomes of the *B. pumilus* reference strain, ATCC 7061^T^ (PRJNA29785), and of strain SF214 (PRJNA290581) and found homologs of most biofilm genes of *B. subtilis* (Table 1). Homologs were also present in other *B. pumilus* strains whose genomes are available on the NCBI databank (CCTCCM205165; SAFR032; S-1; CCMA-560; INR7; BA06). In all cases the similarities with proteins of the strain ATCC 7061^T^ were between 95% and 100% (data not shown). With the exception of the *epsJ* and *epsO* genes*,* not present in either strain of *B. pumilus*, all other genes of the *epsA–O* (Figure 2A) and *tapA-sipW-tasA* (Figure 2B) operons were present and organized as in *B. subtilis*. The function of the products of the *epsJ* and *epsO* genes of *B. subtilis* is not known in detail. It has only been reported that mutations in the *epsJ* gene display a defect in pellicle formation and swarming [19]. The *B. pumilus* strains here considered, both lacking *epsJ*, form a pellicle similar to that of the wild-type strain of *B. subtilis* (not shown)*.* Therefore, the function of the *epsJ* product is still elusive. The gene synteny was similar between the two species, with the *tapA-sipW-tasA* operon adjacent but divergently oriented with respect to the *sinI–sinR* genes, also involved in matrix synthesis (Figure 2B).

**Table 1:** Genes and putative proteins involved in matrix synthesis in *B. subtilis* 168, *B. pumilus* ATCC 7061^T^, and *B. pumilus* SF214.

Genes	Putative Encoded Protein	Protein Identity (%)	
		*B. subtilis* 168 *vs. B. pumilus* ATCC 7061^T^	*B. pumilus* ATCC 7061^T^ *vs. B. pumilus* SF214
**Eps Operon**			
*epsO*	pyruvil transferase	-	-
*epsN*	pyridoxal phosphate-dependent aminotransferase	68	97
*epsM*	acetyl transferase	50	94
*epsL*	sugar transferase	65	97
*epsK*	membrane protein	50	96
*epsJ*	glycosyl transferase	-	-
*epsI*	pyruvil transferase	60	95
*epsH*	glycosyl transferase	46	96
*epsG*	membrane protein	72	96
*epsF*	glycosyl transferase	54	95
*epsE*	glycosyl transferase	61	96
*epsD*	glycosyl transferase	54	94
*epsC*	polysaccharide biosynthesis protein	62	99
*epsB*	tyrosine-protein kinase	63	99
*epsA*	capsular biosynthesis protein	43	100
**TasA Operon**			
*tapA*	lipoprotein for formation	42	93
*sipW*	signal peptidase I	52	98
*tasA*	spore coat protein N	62	97

The identity of the predicted products of the *epsA–O* and *tapA-sipW-tasA* operons varied from 42% to 68% between *B. subtilis* and *B. pumilus*, while proteins of the two *B. pumilus* strains were almost identical (Table 1). TapA, the product of the promoter proximal gene of the *tapA-sipW-tasA* operon, was the least conserved of the biofilm proteins analyzed here. In *B. subtilis*, TapA is required to anchor TasA amyloid fibers to the cell wall [20] and its prominent features are an N-terminal signal secretion sequence, five conserved cysteine residues and an eight amino acid sequence at the N-terminal part of the protein (region 50–57), involved in TasA fiber formation *in vitro* [20]. In both *B. pumilus* strains TapA is shorter than in *B. subtilis* (175 *vs.* 253 amino acids), and the differences were mainly found in the C- and N-terminal regions of the proteins (see Supplementary Materials Figure S1). The 60 C-terminal residues of the *B. subtilis* protein, totally lacking in TapA of *B. pumilus* (see Supplementary Materials Figure S1), are homologous to the SPAM (Secreted Polymorphic Antigen-associated with Merozoites) domains of *Plasmodium falciparum* [21,22] and are not required for TasA fiber formation *in vitro* [20]. At the N-terminal all TapA proteins have signal secretion sequences. Those of *B. pumilus* are not similar to that of *B. subtilis* (see Supplementary Materials Figure S1) but were recognized as signal secretion sequences with a predicted cleavage site at position 30 (see Supplementary Materials Figure S2). TapA of *B. pumilus* has the five cysteine residues conserved in other *Bacillus* species [20], but one of the cysteine residues is within the signal secretion sequence (see Supplementary Materials Figures S1 and S2). The eight amino acids region (50–57) essential for TasA fiber formation *in vitro* in *B. subtilis* [20] is not conserved in *B. pumilus* (Supplementary Materials Figure S1), suggesting either a different mechanism of TapA-mediated anchoring of TasA fiber to the cell wall or a different role of TapA in matrix assembly. The other two products of the *tapA-sipW-tasA* operon, TasA and SipW, are, however, similar in size and primary structure to the two former spore species (see Supplementary Materials Figure S3).

A previous study has reported that the overall similarity of the *eps* operons of *B. subtilis* and *B. cereus* is relatively low and that only the products of *epsC*, *epsL*, and *epsN* genes are conserved [14]. The same three genes are also among the most conserved matrix genes between *B. subtilis* and *B. pumilus* (Table 1), pointing to an essential role of their products in matrix biosynthesis.

### 2.2. Physiological and Chemical Characterization of the Matrix of B. pumilus SF214

**Figure 3:**
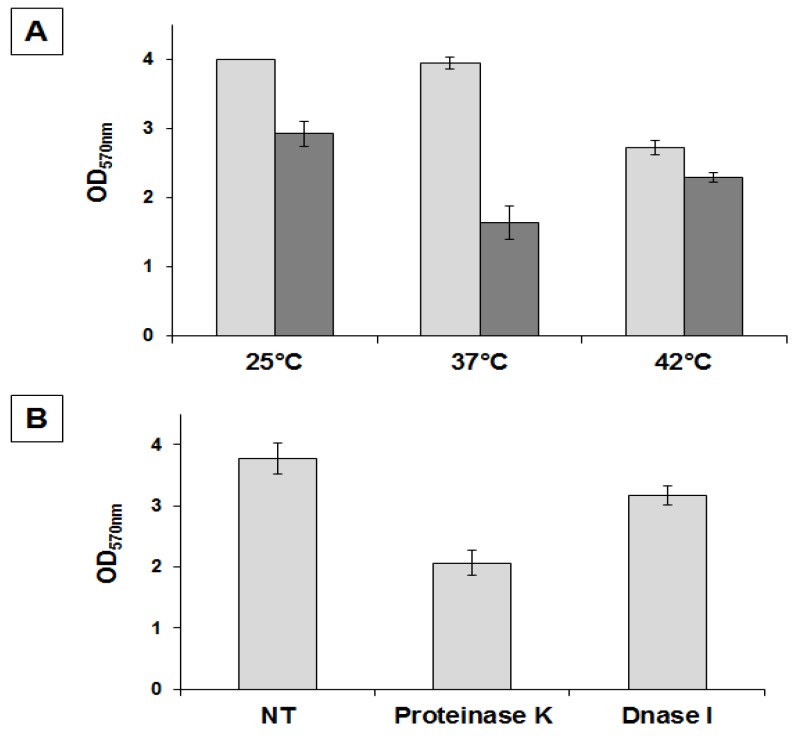
Determination of amount of biofilm produced by *B. pumilus* SF214. (**A**) Cells were grown at the indicated temperatures in rich (LB; light gray bars) or sporulation-inducing (DS; dark gray bars) media for 48 h. Cells were then removed, wells were stained and washed, and the OD (570 nm) was determined. (**B**) Biofilm formation at 25 °C in LB medium supplemented with proteinase K or DNase I, as previously indicated [14].

In liquid cultures of *B. pumilus* SF214, matrix formation is an alternative to sporulation and in some cells parallel to pigment production [18]. In order to better characterize matrix production in strain SF214 we performed an EPS assay (see Section 3.3) on cells grown at different temperatures and in different media. In a rich (LB) medium we observed the highest production of biofilm, similar at 25 and 37 °C and approximately 25% reduced at 42 °C (Figure 3A). In Difco Sporulation (DS) medium we observed the highest production of biofilm at 25 °C, similar at 42 °C and reduced by approximately 30% at 37 °C (Figure 3A). Cells grown in DS medium produced less biofilm than cells grown in LB medium at all tested temperatures (Figure 3A). A reduced production of biofilm in a medium that induces sporulation (DS), known to cause a rapid increase in the cellular concentration of Spo0A–P, is in agreement with the regulatory model previously reported for *B. subtilis* [10] and schematically summarized in Figure 1. We next analyzed the composition of the matrix, investigating whether proteins and/or DNA are important matrix components by preventing their accumulation outside cells with protease and nuclease treatment as previously described for the matrix of *B. cereus* [14]. Proteinase K was more effective at preventing biofilm formation than DNase I, reducing the amount of biofilm by approximately 50% (Figure 3B). This suggests that proteins play an important role in biofilm formation and thus confirms the similarities with *B. subtilis* observed by the bioinformatic analysis.

**Figure 4:**
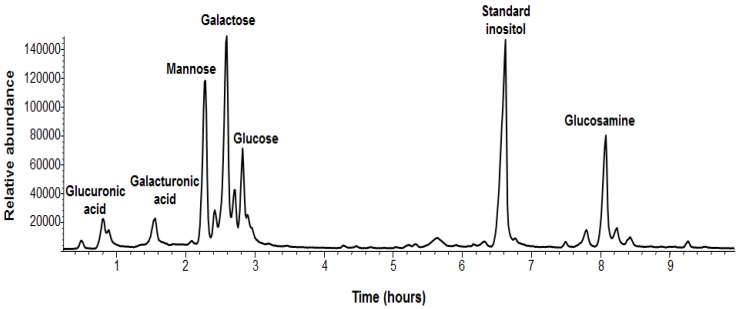
GC-MS chromatogram and assignments of *B. pumilus* SF214 strain biofilm monosaccharides. The non-indicated peaks in the chromatogram are different forms of the same monosaccharide derivative (*i.e.*, α,β-furanosidic and pyranosidic forms).

In addition, we analyzed the chemical composition of the sugars of the EPS of *B. pumilus* SF214 (Figure 4 and Table 2). EPS monosaccharides were analyzed by GC-MS as acetylated *O*-methyl glycosides. The sample was treated with 1.25 M MeOH/HCl and subsequently acetylated with acetic anhydride in pyridine and analyzed by GC-MS. The evaluation of the retention times and the fragmentation pattern from the GC-MS spectra compared with authentic standards allowed the identification of the monosaccharide residues. The sample contained galactose as its major component, together with minor amounts of mannose, 2-deoxy-2-amino glucose (glucosamine), galacturonic acid, and glucuronic acid (Figure 4). The relative abundances of monosaccharides are shown in Table 2.

**Table 2:** Relative abundance of monosaccharides found in the matrix of *B. pumilus* SF214.

Monosaccharide	Relative Abundance ^a^
Galactose	1.00
Mannose	0.75
Glucosamine	0.54
Glucose	0.52
Galacturonic acid	0.21
Glucuronic acid	0.15

^a^ The most abundant monosaccharide is arbitrarily considered as unitary.

### 2.3. Mutants with Altered Pigmentation Are Also Altered in Matrix Formation and Sporulation

**Figure 5:**
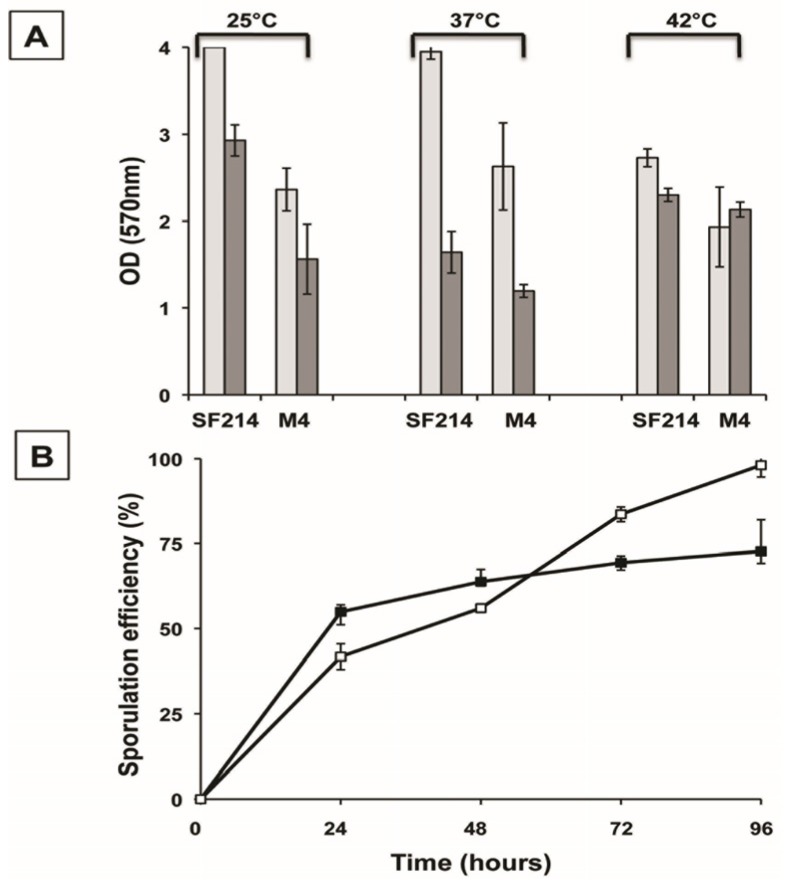
Biofilm formation (**A**) and sporulation efficiencies (**B**) of strain SF214 and M4 mutant. (**A**) Cells were grown at the indicated temperatures in rich (LB; light gray bars) or sporulation-inducing (DS; dark gray bars) media for 48 h. Cells were then removed, wells were stained, washed and the OD (570 nm) determined. (**B**) At the indicated times a sample of each growing culture (SF214 and M4 strain in black and white squares, respectively) was collected and split into two aliquots that were diluted and plated on LB plates. One of the aliquots was heat-treated (20 min at 80 °C) to kill all vegetative cells before plating. For each time point, the sporulation efficiency is the percentage of cells growing after the heat treatment, considering as 100% the number of cells growing without the heat treatment.

**Figure 6:**
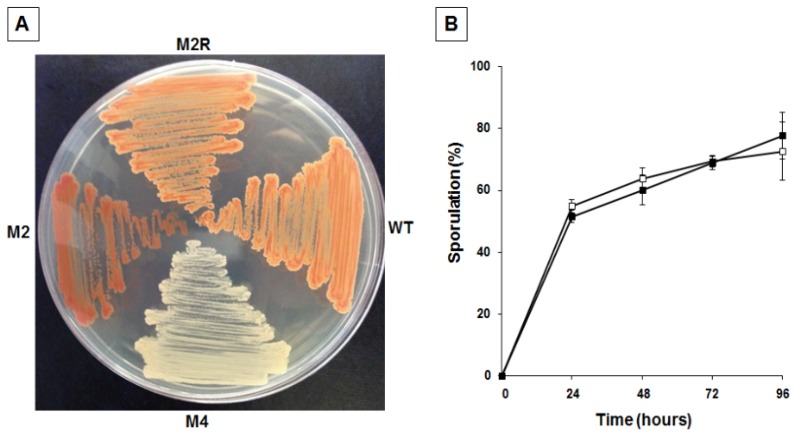
Pigmentation (**A**) and sporulation efficiency (**B**) of wild type and mutants of strain SF214. (**A**) Cells of wild type (wt), unpigmented (M4), over-pigmented (M2) mutants, and spontaneous revertant of M2 (M2R) were grown for 36 h at 25 °C on LB plate. (**B**) At the indicated times a sample of each growing culture (SF214 and M2R strain in black and white squares, respectively) was collected and split into two aliquots that were diluted and plated on LB plates. One of the aliquots was heat-treated to kill all vegetative cells before plating. For each time point, the sporulation efficiency is the percentage of cells growing after the heat treatment, considering as 100% the number of cells growing without the heat treatment.

We have previously reported the isolation of an unpigmented mutant of strain SF214 after nitrosoguanidine (NTG) mutagenesis [18]. One mutant (M4), carrying a single mutation, was used to demonstrate the anti-oxidative activity of the pigment [18]. We used the unpigmented M4 mutant to measure biofilm formation and sporulation. Compared to its wild type (SF214), in the mutant strain (M4) biofilm production at 25 and 37 °C was strongly reduced in a rich (LB) medium, while a lesser reduction was observed at 42 °C (Figure 5A). In a sporulation-inducing (DS) medium the wild type produced more biofilm than the mutant only at 25 °C, while at 37 and 42 °C similar amounts of biofilm were produced by the two strains (Figure 5A). The efficiency of sporulation of SF214 and M4 strains is shown in Figure 5B. Strain M4 showed an efficiency of sporulation similar to its wild-type strain for the first 48 h of growth and a slightly higher sporulation efficiency after 72 and 96 h. The efficiency of sporulation was also measured by counting the free spores under the light microscope at the various time points. Five microscope fields containing at least 200 cells/spores were randomly selected for each strain and cells and phase bright spores counted. Results are shown in Supplementary Materials Figure S4 and were almost identical to those reported in Figure 5B. Those results indicate that the mutation carried by strain M4, in addition to block pigment synthesis ([18]; Figure 6A), also affects sporulation and biofilm formation. Based on this, we hypothesize that the mutation carried by the M4 strain is most likely in a gene coding for a regulatory factor rather than in one of the genes coding for an enzyme of the pigment biosynthetic pathway.

To confirm the link between pigmentation, matrix synthesis, and sporulation, we decided to identify and characterize other mutants with an altered pigmentation. Mid-exponential phase cells were incubated for different times with 10 mg of NTG and the percentage of survival assessed by colony forming unit (CFU) determination, as previously reported [18]. To minimize the possibility of having mutants carrying multiple mutations, only cells exposed to NTG for the shortest time were considered. NTG-treated cells were then diluted, plated, and checked for pigmentation after 36 h of incubation at 25 °C. We focused on a single strongly over-pigmented mutant, M2. As previously reported [18], *B. pumilus* SF214 is refractory to genetic transformation, making it extremely difficult to identify the mutation carried by M2. A first phenotypic characterization of the mutant revealed that it was totally unable to produce spores. This surprising observation induced us to consider the possibility that we had either isolated a contaminant of some other bacterial species or that more than one mutation was carried by the M2 strain. To address these points we first analyzed and compared the DNA sequences of genes coding for the 16S RNA (not shown) and several other genes (see Section 2.4) of SF214 and M2 strains. All M2 genes analyzed were identical to their counterparts in strain SF214 (see Section 2.4), allowing us to exclude that M2 was a contaminant of a different bacterial species. Then, to evaluate whether more than one mutation was carried by the M2 strain we followed a classical genetic approach. We observed that the over-pigmented phenotype reverted spontaneously at a frequency of 1 clone out of 10^9^. Then, we analyzed one revertant, M2R, and observed that it was identical to strain SF214 for its pigmentation (Figure 6A) and for its efficiency in producing spores (Figure 6B). Reversion of both phenotypes at a frequency of one clone out of 10^9^ makes it extremely unlikely that the two phenotypes are due to more than one mutation.

**Figure 7:**
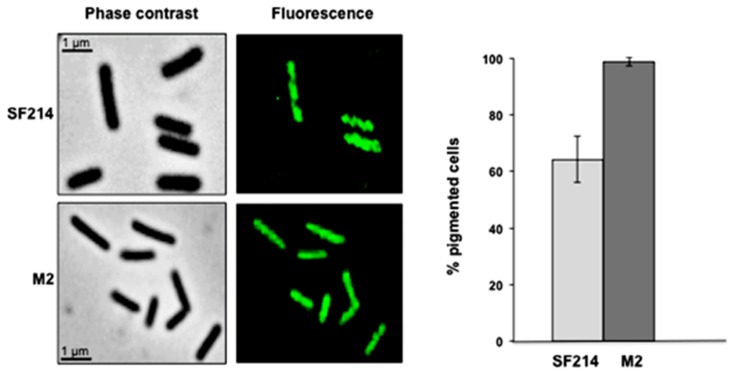
Pigmentation of strain SF214 and its mutant M2. Growing cells of strain SF214 and of the M2 mutant were analyzed by phase contrast and fluorescence microscopy. A representative microscopy field for each strain is shown by phase contrast and fluorescence microscopy. Over 1000 cells of 20 different microscopy fields for each strain were observed and the percentage of fluorescent cells reported.

A fluorescence microscopy analysis of strains SF214 and M2 showed that the number of fluorescent cells of the mutant was strongly increased. Almost all mutant cells were fluorescent at conditions (25 °C in LB medium) in which slightly over 60% of the wild-type cells were pigmented (Figure 7). Pigment production in SF214 is a bimodal process [18] and the number of fluorescent cells is known to vary in response to environmental conditions [18]. The increase in the number of fluorescent cells in M2 is an indication that the mutation carried by the M2 strain is not in a gene coding for a pigment biosynthetic enzyme but rather in a regulatory factor influencing the matrix synthesis/sporulation switch [5].

**Figure 8:**
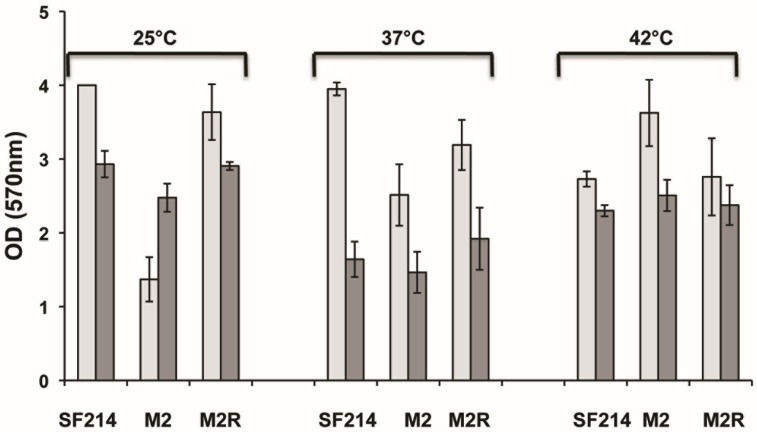
Biofilm formation efficiency of strain SF214, M2 mutant, and M2R revertant. Cells were grown at the indicated temperatures in rich (LB; light gray bars) or sporulation-inducing (DS; dark gray bars) media for 48 h. Cells were then removed, wells were stained and washed, and the OD (570 nm) was determined.

A phenotypic analysis of M2 mutant cells showed that they are thinner (average width of 0.58 ± 0.1 and 0.45 ± 0.08 μm for SF214 and M2 strains, respectively) than wild-type cells. This phenotype has been associated with alteration in the efficiency of biofilm formation in *B. subtilis* [23,24] as well as in other microorganisms [25,26]. To verify whether these phenotypes are also linked to matrix synthesis in *B. pumilus*, we analyzed biofilm synthesis in strains SF214 and M2. As shown in Figure 8, biofilm production was clearly reduced in M2 in a rich (LB) medium at 25 and 37 °C, while at 42 °C the situation was reversed, with the mutant producing more biofilm than strain SF214 (Figure 8). When cells were grown in a sporulation-inducing (DS) medium, wild-type and mutant strains produced similar amounts of biofilm at all three temperatures tested (Figure 8). In the experiments shown in Figure 8 we also analyzed the amount of biofilm produced by the revertant strain M2R, which, as expected, behaved like the wild-type strains at all conditions tested (Figure 8). Experiments shown in Figure 8 then suggest that the observed changes in cell morphology are linked to a defect in matrix synthesis in the M2 mutant. In conclusion, the mutation carried by M2 increased pigment synthesis, blocked sporulation, and affected biofilm formation. Therefore, as in the case of the M4 mutant reported above, for the M2 mutant it is also more likely that the mutation is in a gene coding for regulatory factor rather than in one of the genes of the pigment biosynthetic pathway.

### 2.4. In Search of the Mutations

Due to the difficulties in manipulating strain SF214, the availability of genome data, and the possibility that the mutation carried by each of the two mutants could be in one of the regulatory factors determining the switch between sporulation and matrix production, we decided to analyze the DNA sequences of the genes coding for the main regulators controlling the spore/matrix switch indicated in Figure 1. The analysis of strain SF214 genome showed that it contains homologs of the main regulators identified in *B. subtilis* (Table 3). The identity of the predicted products of the genes analyzed varied from 42% to 93% between *B. subtilis* and *B. pumilus*, while proteins of the two *B. pumilus* strains were almost identical (Table 3). Of the analyzed gene products, SinI and Spo0B are the least conserved ones between the two species (Table 3).

**Table 3:** Genes and putative proteins involved in the regulation of matrix synthesis and sporulation in *B. subtilis* 168, *B. pumilus* ATCC 7061^T^, and *B. pumilus* SF214.

Genes	Putative Encoded Protein	Protein Identity (%)	
		*B. subtilis* 168 *vs.* *B. pumilus* ATCC 7061^T^	*B. pumilus* ATCC 7061^T^ *vs.* *B. pumilus* SF214
*sinI*	sinR antagonist	45	96
*sinR*	transcriptional regulator	93	98
*spo0A*	sporulation protein A	88	100
*spo0B*	phosphotransferase B	58	98
*spo0F*	phosphotransferase F	92	100

All genes indicated in Table 3 were sequenced in both M2 and M4 mutants (data not shown). No differences were identified in any of the genes analyzed, indicating that none of these genes was responsible for the observed phenotypes. These negative results indicate that other factors are involved in or influence the fate of stationary cells of the SF214 strain. These could either be *B. pumilus* SF214-specific proteins or homologs of proteins characterized in *B. subtilis* as affecting biofilm synthesis but not analyzed in this study. These include AbrB [10], RemA-B [27,28], Veg [29], and SlrA [30], and also proteins only indirectly related to biofilm, such as collagen-like proteins [31], components of the RapP-PhrP quorum sensing system [32], and ribosomal proteins S11 and S21 [33]. In addition, mutations in genes coding for alpha-phosphoglucomutase [23], CcpA, glutamate synthase, GltAB, and the aminopeptidase AmpS [34] have all been shown to have pleiotropic effects and also influence biofilm formation. All those genes are potential targets of the mutations carried by strains M2 and M4, making unrealistic the sequence-based approach to identify the mutated gene(s). In conclusion, the factor(s) controlling the biofilm-matrix switch in SF214 remains elusive and its identification is a challenging future task that will necessarily require the development of genetic tools to manipulate this pigmented marine microorganism.

## 3. Experimental Section

### 3.1. Bacterial Strains and Growth Conditions

*Bacillus subtilis* (PY79) [35], *B. pumilus* ATCC 7061^T^, and *B. pumilus* SF214 strain [17] were grown either in LB medium (for 1 L: 10 g Bacto-Tryptone, 5 g Bacto-yeast extract, 10 g NaCl, pH 7.0) or in Difco Sporulation (DS) medium in aerobic conditions at 25, 37, or 42 °C.

### 3.2. Fluorescence Microscopy

For autofluorescence 200 µL aliquots of cell culture were centrifuged (2 min, 6000 *g*) and cells resuspended in 20 µL of phosphate-buffered saline (PBS, pH 7.4). Six microliters of each sample were placed on microscope slides and covered with a coverslip previously treated for 30 s with poly-l-lysine (Sigma, St.Louis, MO, USA). Poly-l-lysine (PLL) is a cationic polymer used for attaching and immobilizing cells to glass substrates since it increases the number of positively-charged sites available for cell binding and promotes electrostatic interactions with negatively-charged ions of bacterial cell surface. Samples were observed with an Olympus BX51 fluorescence microscope (Olympus, Hicksville, NY, USA) using a Fluorescein-Isothiocyanate (FITC) filter to visualize the fluorescence of the cells. Typical acquisition times were 1000 ms for autofluorescence and the images were captured using a Olympus DP70 digital camera (Olympus, NY, USA) and processed.

### 3.3. Biofilm Production Assay

To test biofilm production, overnight cultures were used to inoculate liquid LB and DS medium and cells grown at 25 °C, 37 °C, and 42 °C in static conditions for up to 48 h. When required, LB medium was supplemented with proteinase K (0.1 mg/mL) (Qiagen, Milan, Italy) or DNaseI (5 U/mL) (Qiagen, Milan, Italy) to assess whether proteins and/or extracellular DNA are important components of the biofilm matrix. Protease and nuclease treatments prevent the accumulation of proteins and DNA, respectively, outside the cell. The medium was removed and the wells (35-mm culture dish) were washed five times with dH_2_O to remove loosely associated bacteria. Plates were air-dried for 30 min and each well was stained for 30 min with 1 mL 0.1% (wt/vol) crystal violet in an isopropanol-methanol-PBS solution (1:1:18 (vol/vol)). Crystal violet will bind primarily to negatively-charged molecules of biofilm biomass (cell surface, extracellular DNA, and EPS) allowing the assessment of the biofilm produced [13]. After staining, the wells were washed with dH_2_O and air-dried (about 30 min). The crystal violet bound was extracted with an ethanol-acetone (80:20) solution and the optical density (OD) of each sample was measured at 570 nm.

### 3.4. EPS Extraction

After development of a mature biofilm, 60 µL of formaldehyde (36.5% solution) were added to each 10 mL of sludge to fix the cells and prevent cell lysis during subsequent steps. The formaldehyde-sludge mixture was incubated at room temperature in a fume cabinet with gentle shaking (100 rpm) for 1 h. Four milliliters of 1 M NaOH were added for each 10 mL of sludge and the resulting mixture was incubated at room temperature with shaking for 3 h to extract EPS. Cell suspensions were then centrifuged (16,800× *g*) for 1 h at 4 °C. The supernatant containing soluble EPS was filtered through a 0.2-µm filter (Corning, Cambridge, MA, USA, ) and dialyzed against distilled water using a 12–14 kDa molecular weight cut-off (MWCO) membrane (SERVA, Milan, Italy) for 24 h at 25 °C.

### 3.5. Purification of Exopolysaccharides

Trichloroacetic acid (TCA) was added (20% w/v) to extracted EPS solutions on ice to precipitate proteins and nucleic acids. After 30 min, the solution was centrifuged (16,800× *g*) for 1 h at 4 °C, the supernatant was collected, 1.5 volumes of 95% ethanol were added, and the mixture was placed at −20 °C for 24 h to precipitate exopolysaccharides away from lipids. The solution was then centrifuged (16,800× *g*) for 1 h at 4 °C and the exopolysaccharide pellet was resuspended in Milli-Q water and dialyzed against the same for 24 h at 4 °C using a 12–14 kDa MWCO membrane to remove low molecular weight impurities. The remaining retentate was lyophilized overnight.

### 3.6. Carbohydrate Compositional Analysis

Carbohydrates were analyzed as previously described [36]. Briefly, the sample (1 mg) was dried and methanolyzed by adding 1.25 M HCl in MeOH (0.5 mL) for 20 h at 80 °C. After methanol evaporation, it was acetylated with acetic anhydride (25 μL) and pyridine (50 μL) at 80 °C for 30 min and, after work-up, analyzed by GC-MS for carbohydrate quali-quantitative analysis.

All the analyses were performed with an Agilent instrument (GC instrument Agilent 6850 coupled to MS Agilent 5973) (Agilent Technologies, Santa Clara, CA), equipped with a SPB-5 capillary column (Supelco, 30 m × 0.25 mm i.d., flow rate, 0.8 mL·min^−1^)) and helium as carrier gas. Electron impact mass spectra were recorded with an ionization energy of 70 eV and an ionizing current of 0.2 mA. The temperature program used for the analyses was the following: 150 °C for 5 min, increased to 280 °C at 3 °C/min, 300 °C for 5 min.

### 3.7. DNA Extraction and PCR Procedure

Chromosomal DNA from *B. pumilus* was isolated as described previously for *B. subtilis* [37]. Briefly, exponentially growing cells were centrifuged at 7000 rpm for 10 min and the pellet resuspended in lysis buffer (50 mM EDTA, 100 mM NaCl). Two point five milligrams per milliliter of lysozyme were added and the mixture was incubated at 37 °C for 30 min without shaking. Then, sarcosyl 20% (w/v) was added and re-incubated at 37 °C for 10 min. Chromosomal DNA was extracted with 1 volume of phenol and precipitated in the recovered aqueous phase by adding 1/10 volume of sodium acetate (3 M pH 5.2) and 2.5 volumes of cold ethanol. Amplification reactions (PCR) were performed by using chromosomal DNA as a template and synthetic oligonucleotides (see Supplementary Materials Table S1) as primers. Nucleotide sequences of the amplified genes were performed externally (BMR Genomics, Padova, Italy).

### 3.8. Bioinformatic Analysis

To find regions of similarity between nucleotide or protein sequences all the alignments were performed using the Basic Local Alignment Search Tool (BLAST) [38]. Protein sequences of strain SF214 were aligned against all protein sequences of *B. subtilis* 168 and *B. pumilus* strains from NCBI database. The results with the best E-score, coverage and identity percentage were considered for our study. Genome assembly of *Bacillus pumilus* SF214 was performed with MIRA v4.0.2 and the contigs were mapped *versus* the publically available genomes of *B. pumilus* retrieved from NCBI database. Then, Prodigal v2.6 was used for open reading frame prediction and automatic annotation of the ORFs was performed using BLAST to align identified *B. pumilus* SF214 ORFs against NCBI databases.

## 4. Conclusions

SF214 is a pigmented strain of *Bacillus pumilus* isolated from the marine environment, able to form an extracellular matrix composed of proteins and carbohydrates. Galactose together with minor amounts of mannose, glucosamine, galacturonic acid, and glucuronic acid represents the carbohydrate portion of the matrix. The growth temperature and the composition of the growth medium control the amount of extracellular matrix produced by cells of the SF214 strain. In this marine bacterium matrix synthesis relies on orthologs of many of the genes known to be important for matrix synthesis in *B. subtilis*, the model system for spore-forming *Bacilli*. Mutants of SF214 characterized by an altered pigmentation were also altered in the amount of matrix produced and in the efficiency of spore formation. This observation suggests that in this strain of *B. pumilus* pigment formation, matrix synthesis and sporulation are interconnected processes, regulated by a common mechanism. The molecular details of the genetic switch responsible of controlling the three processes is still in progress.

## Supplementary Files

Supplementary File 1
