# Supplementary material for: Matrix Production, Pigment Synthesis, and Sporulation in a Marine Isolated Strain of Bacillus pumilus
Source: Mar Drugs. 2015 Oct 21;13(10):6472–88. doi: 10.3390/md13106472 (PMC4626701; doi:10.3390/md13106472)
Supplement: Supplementary File 1 [file marinedrugs-13-06472-s001.docx]

Supplementary Materials


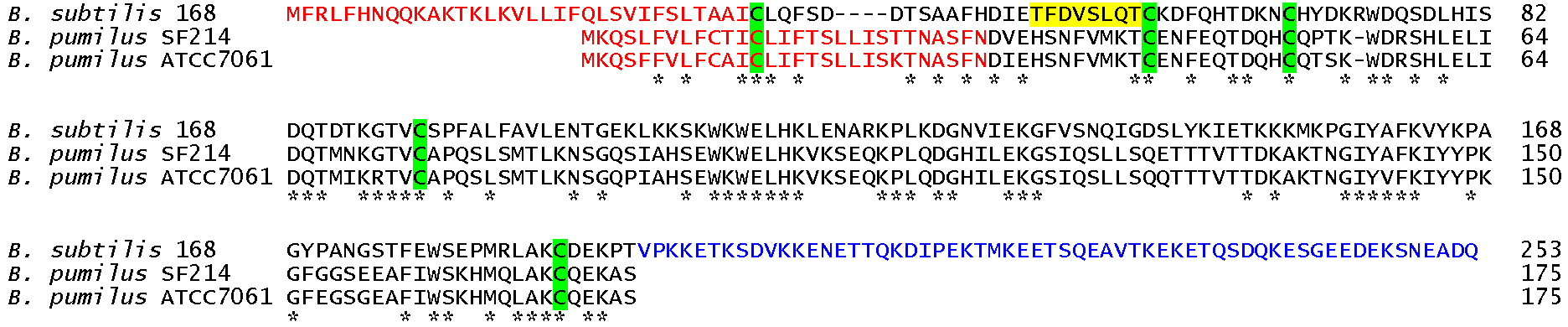


**Figure S1.** Alignment of TapA proteins of *B. subtilis* 168, *B. pumilus* SF214, and *B. pumilus* ATCC 7061T. Asterisks indicate identical residues. In red are the N-terminal signal secretion sequences [1] and Figure. S2 for *B. subtilis* and *B. pumilus*, respectively). In blue is a 60-amino acid region of TapA of *B. subtilis* lacking in the *B. pumilus* proteins. The region in blue is homologous to SPAM (Secreted Polymorphic Antigen-associated with Merozoites) domains typically found in *Plasmodium falciparum* [2,3]. Highlighted in green are five conserved cysteine residues considered a prominent feature of TapA of
*B. subtilis* [4] Highlighted in yellow is a region of TapA of *B. subtilis* (50–57) essential for allowing TasA fiber formation [4] that is not conserved in *B. pumilus*.


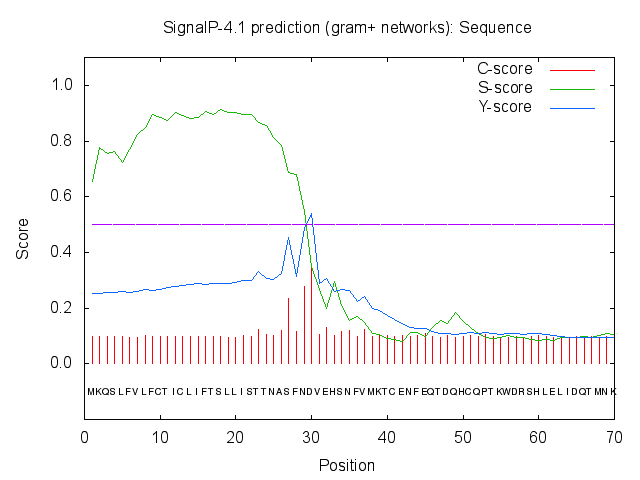


**Figure S2.** Bioinformatic prediction of signal secretion sequences in TapA of *B. pumilus*. The signal sequence (mean S-score: 0.823_position: 1–29) and signal peptidase cleavage sites (max C-score: 0.357_position: 30; max Y-score: 0.541_position: 30) of TapA of
*B. pumilus* were predicted by the SignalP 4.0 program.


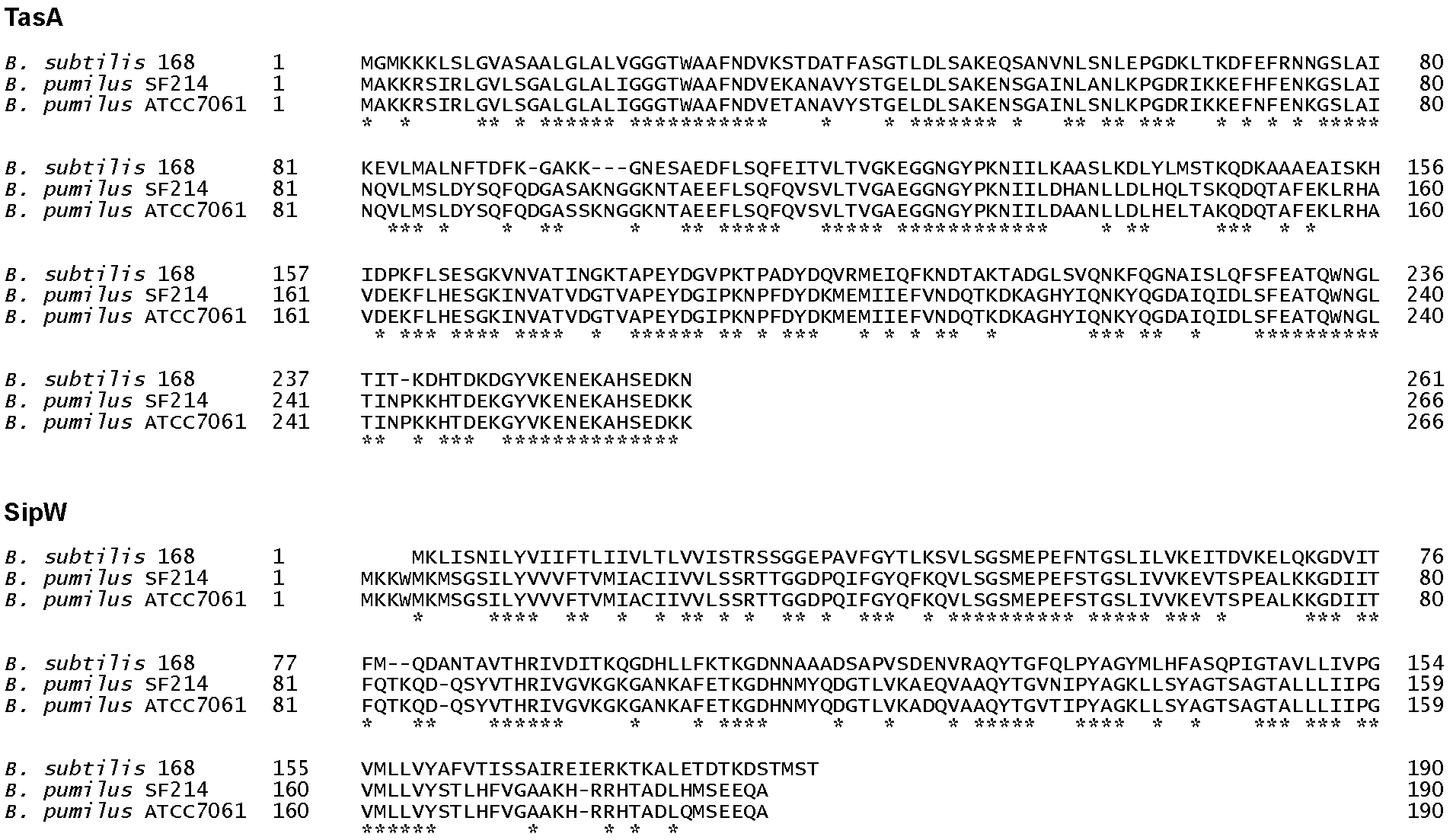


**Figure S3.** Alignment of TasA and SipW proteins of *B. subtilis* 168, *B. pumilus* SF214, and *B. pumilus* ATCC 7061T. Asterisks indicate identical residues.

**Figure S4.** Efficiency of sporulation of strains SF214 and M4. Five microscope fields were randomly selected for each strain and about 200 cells/spores counted for each field. The sum of spores and vegetative cells was considered as 100% for each time point and strain.

**Table S1.** Synthetic oligonucleotides used.

| **Gene** | **Primer Sequence** | **Amplicon** |
| --- | --- | --- |
| *spo0A* | spo0A_BPUF 5′- AGAAAAAGCTAGATAATGCTC-3′  spo0A_BPUR 5′-GATGATTGTATCAATACAACC-3′ | 1026 bps |
| *spo0F* | spo0F_BPUF 5′-GACTGATTTCCTCCTTCGG-3′  spo0F_BPUR 5′-CTCTCCACCCTAGCATGAC -3′ | 688 bps |
| *spo0B* | spo0B_BPUF 5′- GAACGTTTGCTGACTTTGCC -3′  spo0B_BPUR 5′-CACGACGGAACGCCACC-3′ | 850 bps |
| *sinI-sinR* | sinFor 5′-CCCATAGGTCCCCTCCC-3′  sinRev 5′-GCTCTGCATAGCAATTGGAG-3′ | 759 bps |
| 16S | Ribo-For 5′-AGTTTGATCCTGGCTCAG-3′  Ribo-Rev 5′-CCTACGTATTACCGCGGC-3′ | 540 bps |

References

1. Branda, S.S.; Gonzalez-Pastor, J.E.; Ben-Yehuda, S.; Losick, R.; Kolter, R. Fruiting body formation by *Bacillus subtilis*. *Proc. Natl. Acad. Sci. USA* **2001**, *98*, 11621–11626.
2. McColl, D.J.M.; Silva, A.; Foley, M.; Kun, J.F.; Favaloro, J.M.; Thompson, J.K.;
   Marshall, V.M.; Coppel, R.L.; Kemp, D.J.; Anders, R.F. Molecular variation in a novel polymorphic antigen associated with *Plasmodium falciparum* merozoites. *Mol. Biochem. Parasitol.* **1994**, *68*, 53–67.
3. Mulhern, T.D.; Howlett, G.J.; Reid, G.E.; Simpson, R.J.; McColl, D.J.; Anders, R.F.; Norton, R.S. Solution structure of a polypeptide containing four heptad repeat units from a merozoite surface antigen of *Plasmodium falciparum*. *Biochemistry* **1995**, *34*, 3479–3491.
4. Romero, D.; Vlamakis, H.; Losick, R.; Kolter, R. Functional analysis of the accessory protein TapA in *Bacillus subtilis* amyloid fiber assembly. *J. Bacteriol.* **2014**, *196*, 1505–1513.

© 2015 by the authors; licensee MDPI, Basel, Switzerland. This article is an open access article distributed under the terms and conditions of the Creative Commons Attribution license (http://creativecommons.org/licenses/by/4.0/).
